# Clinical outcomes of single-stage versus two-stage laparoscopic Roux-en-y gastric bypass in the management of obesity (BMI ≥ 50 kg/m^2^): a retrospective cohort study

**DOI:** 10.1007/s00423-022-02664-9

**Published:** 2022-09-02

**Authors:** Michael G Fadel, Matyas Fehervari, Ali Lairy, Bibek Das, Khaled Alyaqout, Hutan Ashrafian, Haris Khwaja, Evangelos Efthimiou

**Affiliations:** 1 Department of Bariatric and Metabolic Surgery, Chelsea and Westminster Hospital, London, UK; 2 Imperial College London, London, UK; 3 Mubarak Al-Kabeer Hospital, Jabriya, Kuwait; 4 Jaber Al-Ahmad Al-Sabah Hospital, Kuwait City, Kuwait

**Keywords:** Bariatric surgery, Obesity, Intra-gastric balloon, Roux-en-Y gastric bypass, Weight loss

## Abstract

**Background:**

Laparoscopic Roux-en-Y gastric bypass (LRYGB) in patients with obesity, BMI ≥ 50 kg/m^2^, can be a challenging operation. Weight loss with intra-gastric balloon (IGB) insertion prior to LRYGB may improve operative outcomes.

**Methods:**

Between June 2000 and June 2020, patients with a BMI ≥ 50 kg/m^2^ underwent either IGB insertion followed by LRYGB (two-stage group), or LRYGB as the definitive bariatric procedure (single-stage group) in our institution. The two-stage procedure was adopted for high risk individuals. Primary outcome measures were percentage total weight loss (%TWL) at 24 months, length of stay and postoperative morbidity. Propensity score analysis was used to account for differences between groups.

**Results:**

A total of 155 (mean age 42.9 years ± 10.60; mean BMI 54.6 kg/m^2^ ± 4.53) underwent either the two-stage (*n* = 30) or single-stage procedure (*n* = 125) depending on preoperative fitness. At 6 months following LRYGB, there was a significant difference in %TWL between the groups in a matched analysis (11.9% vs 23.7%, *p* < 0.001). At 24 months, there was no difference in %TWL (32.0% vs 34.7%, *p* = 0.13). Median hospital stay following LRYGB was 2.0 (1-4) days with the two-stage vs 2.0 (0-14) days for the single-stage approach (*p* = 0.75). There was also no significant difference in complication rates (*p* = 0.058) between the two groups.

**Conclusions:**

There was no difference in weight loss after one or two-stage procedures in the treatment of patients with a BMI ≥ 50 kg/m^2 ^super obesity in a propensity score weighted analysis at 24 months. Length of stay and perioperative complications were similar for high risk patients; however, the two-stage approach was associated with delayed weight loss. Single-stage management is recommended for moderate risk patients, particularly with significant metabolic disorders, whilst two-stage approach is a safe and feasible pathway for high risk individuals.

**Supplementary Information:**

The online version contains supplementary material available at 10.1007/s00423-022-02664-9.

## Introduction

Obesity, body mass index (BMI) ≥ 50 kg/m^2^, has risen more rapidly in the last two to three decades compared to other BMI categories [1]. It is associated with severe morbidity such as hypertension, dyslipidaemia, diabetes mellitus, congestive heart failure and cancer, and has been shown to reduce life expectancy by 9.8 years compared to normal BMI [2]. This in turn poses significant surgical and anaesthetic risks as well as major challenges to the healthcare system [3, 4].

Bariatric metabolic surgery is the most effective method in permanently reducing weight leading to a decrease in all-cause morbidity and mortality in individuals with obesity [5–9]. The most common bariatric procedures for this high risk group of patients include laparoscopic sleeve gastrectomy (LSG) and laparoscopic Roux-en-Y gastric bypass (LRYGB) followed by intra-gastric balloon (IGB) [10]. It has been demonstrated that LRYGB is associated with better long-term weight loss in patients with morbid obesity (BMI ≥ 40 kg/m^2^), in comparison with LSG or laparoscopic adjustable gastric band surgery [11]. However, LRYGB is often challenging both from a surgical and anaesthetic point of view in patients with BMI ≥ 50 kg/m^2^ and is associated with significant perioperative risks [12–17]. The optimum surgical procedure or treatment pathway has yet to be established in the management of these patients.

IGBs are restrictive devices that occupy space in the stomach, delay gastric emptying and effectively reduce liver size [18, 19]. Insertion of IGB is a less invasive, reversible procedure with fewer perioperative risks compared to laparoscopic or open surgery. However, it only provides a temporary solution as significant weight regain has been demonstrated following removal of these devices [20]. Nevertheless, insertion of IGB has the ability to reset patient’s weight and reduce preoperative surgical risks due to less intra-abdominal adipose tissue. An obvious solution to achieve prolonged weight loss following IGB is to offer patients a definitive second stage bariatric procedure, in theory offering sustainable weight loss and simultaneous reduction of perioperative risks. Early experience suggests that IGBs are a useful bridging precursor to definitive bariatric surgery by providing appropriate short-term weight loss by resetting patient’s weight to a lower starting point and improving their perioperative risks before a LRYGB is performed [21].

There is no study directly comparing single-stage LRYGB to multi-stage IGB followed by LRYGB pathways for the treatment of patients with BMI ≥ 50 kg/m^2^. The aim of this study is to report on two-year weight loss and clinical outcomes in high risk patients undergoing two-stage procedures and moderate risk patients managed with a LRYGB alone in a tertiary bariatric centre.

## Methods

### Study design and participants

A review of a prospectively collected bariatric database of consecutive patients with BMI ≥ 50 kg/m^2 ^treated with a single and two-stage approach, in our institution, between June 2000 and June 2020. All patients were assessed preoperatively by a bariatric multidisciplinary team (MDT) which included bariatric surgeons, anaesthetists, dieticians and psychologists. Four bariatric surgeons operated on all cases with each surgeon using the same standardised protocol. All patients were American Society of Anesthesiologists (ASA) grade 3 and above, and therefore underwent consultant-led preoperative anaesthetic clinics. The decision made to treat with a single or two-stage approach was based on the patient’s BMI and whether they were deemed high risk based on surgical and anaesthetic review. High risk features included male sex, obstructive sleep apnoea (OSA), asthma, ischaemic heart disease and features of difficult airway. The intention of the two-stage approach was to improve patient’s health status for definitive surgery and to improve overall long-term weight loss, particularly in those patients who were deemed high risk. The study was approved by the Research and Development office at our institution (Protocol number: PCD906) and registered at clinicaltrials.gov (NCT0514601). This work has been reported in line with the STROBE statement [22].

During the study period, a total of 155 patients underwent either a two-stage procedure consisting of endoscopic IGB followed by LRYGB or a single-stage procedure of LRYGB. Patients of any age group were included. The operative technique used for IGB has been previously described by Ashrafian et al. [21] In summary, IGBs were inserted under a short general anaesthetic. A pre-rolled silicone Orbera (Apollo Endosurgery) balloon was inserted into the stomach and the endoscope was re-introduced in order to place the balloon at the fundus. The balloon was then inflated to a fixed volume of 500 ml of saline mixed with 10 ml of 2% methylene blue. The balloon was removed 6 months after insertion and patients were offered definitive surgery in the form of LRYGB at that point. LRYGB was performed 6 weeks following IGB removal to allow for any possible gastritis to settle. The same balloon and operative technique were used throughout the entire study period.

The technique of hand-sewn anastomosis in LRYGB used in this study has been previously described by Fehervari et al. [23] and is summarised here. The pouch was constructed in a vertical fashion along the lesser curve area using a 34 Fr sizer (11 mm diameter) tube and was 10–20 ml in volume. The length of the pouch was measured at 7 cm from the oesophagogastric junction and was 2 cm in width, and the stomach was transected using stapling devices. The gastro-jejunal anastomosis was constructed hand-sewn in an end-to-side configuration in two continuous layers over the 34 Fr orogastric tube for calibration resulting in an anastomosis diameter of 11–12 mm. The biliopancreatic limb was measured to 60 cm and the alimentary limb to 100 cm. The jejuno-jejunostomy was constructed side-to-side with a staple gun and the defect was closed in a continuous single layer.

### Clinical data evaluated

Patient demographics including age, gender, height, weight and BMI were recorded. Smoking history and the presence of diabetes mellitus, hypertension, dyslipidaemia, OSA, asthma and ischaemic heart disease were recorded. Weight loss following LRYGB for either two-stage or single-stage procedures was recorded at clinical time points of 3, 6, 12 and 24 months. The percentage of total weight loss (%TWL) was calculated by the formula: [(initial weight − follow-up weight)/initial weight] × 100 [24]. Hospital length of stay and postoperative complications categorised by the Clavien-Dindo (CD) classification [25, 26] were also noted in both groups.

### Statistical analysis

Baseline characteristics and outcome variables were compared using Welch’s *t* test or Mann *U* Whitney for continuous variables as appropriate, and Pearson’s chi-square or Fisher exact test for categorical variables. Data was presented as means with standard deviation (SD) or median with range values for continuous variables and frequency with percentage for categorical variables.

A propensity score algorithm was used to balance treatment groups to adjust for baseline differences and to reduce selection bias given the non-randomised nature of this study. Propensity scores were generated using a multivariable logistic regression model. Model co-variates included age, gender, baseline weight prior to surgery and co-morbidities. Matching weights were assigned to patients based on their propensity scores to balance co-variates between groups. This method permits inclusion of all patients and does not require selecting a caliper. The balance of the matched sample was confirmed by checking standardised mean differences (SMD < 10%).

The primary outcome variable was %TWL between treatment groups at the final time point (*p* values of < 0.05 were taken to indicate statistical significance). Secondary outcomes were length of stay following LRYGB and postoperative complications. Weight loss results in the two-stage and single-stage procedure group were tabulated and presented graphically. All statistical analyses were done using the statistical package R software version 4.0.2.

## Results

### Baseline patient characteristics

The mean age of the 155 patients was 42.9 years (SD ± 10.6) with 122 (78.7%) patients being female. The mean preoperative weight was 153.8 kg (SD ± 22.9) with a mean BMI of 54.6 kg/m^2^ (SD ± 4.5). Thirty (19.4%) patients underwent a two-stage procedure of endoscopic IGB followed by LRYGB, and 125 (80.6%) patients had a single-stage procedure of LRYGB. The mean actual weight at LRYGB for the two-stage group was 156.0 kg (SD ± 19.2). The preoperative characteristics of the patients that underwent the two-stage or single-stage procedure are summarised in Table 1. Prior to weight loss intervention, high risk features such as male sex, cardiovascular and respiratory comorbidities were more common in the two-stage group, whilst diabetes registered with a higher frequency in the single-stage group. High risk patients had a higher initial weight (177.2 kg vs 148.2 kg) and BMI (61.9 kg/m^2^ vs 53.4 kg/m^2^). Balance assessment revealed well-matched groups after propensity score weighting (SMD < 0.1 in all groups). The balance plot is shown in Fig. S1.

**Table 1:** Baseline characteristics of the patient population for the two-stage (intra-gastric balloon followed by laparoscopic Roux-en-Y gastric bypass) and single-stage (laparoscopic Roux-en-Y gastric bypass only) procedure including age, gender, diabetes mellitus, hypertension, dyslipidaemia, smoking, initial weight and body mass index

	Two-stage (*n* = 30)	Single-stage (*n* = 125)	*p *value
Mean age, years (SD)	40.7 (10.5)	43.4 (10.6)	0.21
Female (%)	18 (60.0)	104 (83.2)	0.011*
Diabetes mellitus (%)	6 (20.0)	31 (24.8)	0.57
Hypertension (%)	13 (43.3)	39 (31.2)	0.45
Dyslipidaemia (%)	4 (13.3)	14 (11.2)	0.48
Obstructive sleep apnoea (%)	9 (30.0)	22 (17.6)	0.13
Asthma (%)	8 (27.7)	21 (16.8)	0.30
Ischaemic heart disease (%)	6 (20.0)	11 (8.8)	0.10
Number of co-morbidities/patient	1.5	1.1	
Initial mean weight, kg (SD)	177.2 (23.0)	148.2 (19.1)	< 0.001*
Initial mean BMI, kg/m^2^ (SD)	61.9 (6.0)	53.4 (3.3)	< 0.001*
Mean operative time, min (SD)	129 (51.1)	126 (37.0)	0.75
Median length of stay, days (range)	2.0 (1–4)	2.0 (0–14)	0.75

*BMI* body mass index, *SD* standard deviation

^*^Statistically significant

### Weight loss outcomes

**Fig. 1:**
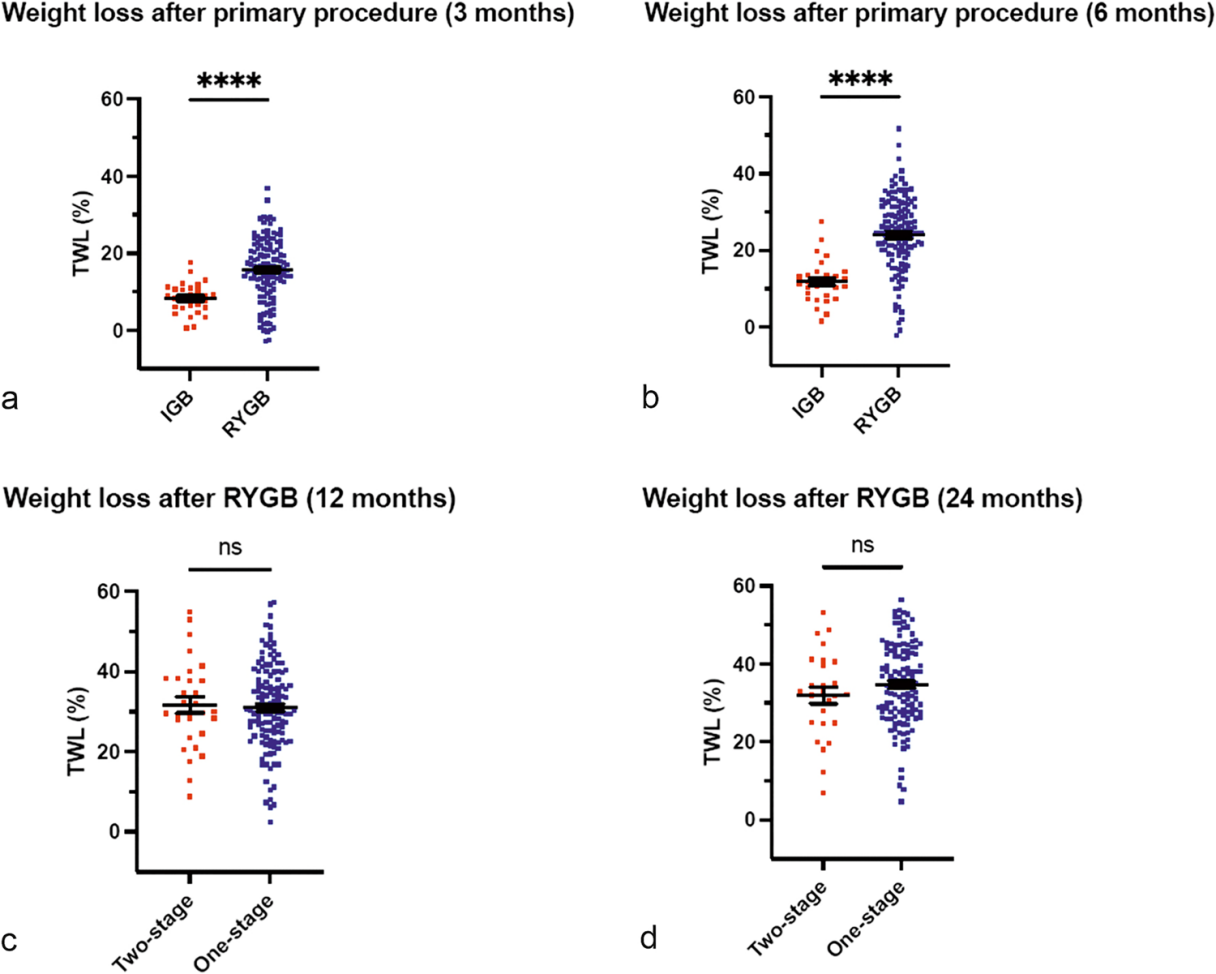
Scatterplot diagrams of percentage total weight loss results after two-stage procedure (intra-gastric balloon followed by laparoscopic Roux-en-Y gastric bypass) and one-stage procedure (laparoscopic Roux-en-Y gastric bypass only) at 3 (**a**), 6 (**b**), 12 (**c**) and 24 (**d**) months. *IGB* intra-gastric balloon, *RYGB* Roux-en-Y gastric bypass, *%TWL* percentage total weight loss, **** statistically significant, *ns* nil significant

One hundred and fifty-five from 201 (77.1%) patients had complete data for each clinical time point following LRYGB during the two-year follow-up period. The %TWL after single-stage and two-stage procedures at 3, 6, 12 and 24 months after LRYGB are displayed in Table 2. Unadjusted *p* values and adjusted *p* are also shown. At 3 and 6 months, the single-stage group had significantly greater %TWL when compared with the two-stage group (15.7% vs 8.3% and 23.7% vs 11.9% respectively, both unadjusted and adjusted *p* < 0.001). There was no difference in weight loss outcomes between the two groups at 12 and 24 months (31.0% vs 31.7% and 34.7% vs 32.0% respectively, both unadjusted and adjusted *p* > 0.05). The %TWL following the single-stage or two-stage procedure at 3, 6, 12 and 24 months are graphically represented in Fig. 1a–d.

**Table 2:** Percentage total weight loss results, unadjusted and adjusted data, after LRYGB for the two-stage (intra-gastric balloon followed by laparoscopic Roux-en-Y gastric bypass) and single-stage (laparoscopic Roux-en-Y gastric bypass only) procedure at 3, 6, 12 and 24 months

Total weight loss post-LRYGB procedure, %	Two-stage (*n* = 30)	Single-stage (*n* = 125)	Unadjusted *p* value	Adjusted *p* value
3 months (SD)	8.3 (3.9)	15.7 (8.1)	< 0.001*	< 0.001*
6 months (SD)	11.9 (5.6)	23.7 (11.4)	< 0.001*	< 0.001*
12 months (SD)	31.7 (11.0)	31.0 (10.9)	0.76	0.43
24 months (SD)	32.0 (11.0)	34.7 (10.7)	0.25	0.13

*LRYGB* laparoscopic Roux-en-Y gastric bypass, *SD* standard deviation

^*^Statistically significant

### Length of hospital stay and postoperative complications

There was no significant difference in mean operative time of LRYGB following IGB (129 ± 51.1 min) when compared to LRYGB only (126 ± 37.0 min, *p* = 0.75). There was also no difference in median length of stay following LYRGB when comparing the two-stage versus single-stage group (2.0 vs 2.0 days, *p* = 0.75).

There were 7 (23.3%) complications in total reported in the two-stage group. There were four IGB complications which included excessive vomiting (*n* = 3) and hospital-acquired pneumonia (*n* = 1). One patient died following IGB insertion as a result of ventricular tachycardia. There were two complications following LRYGB in this group — bleeding (*n* = 1), tight gastro-jejunal anastomosis and perforated marginal ulceration (*n* = 1). In the single-stage group, there was a total of 13 (10.4%) complications recorded including hospital-acquired pneumonia (*n* = 2), postoperative bleeding (*n* = 1), marginal ulceration (*n* = 2) and development of anastomotic strictures (two out of six patients required stricture dilatation). Two other patients returned to theatre due to a collection anterior to the gastro-jejunal anastomosis as a result of a contained leak and obstruction at jejuno-jejunal anastomosis requiring refashioning. Clinically significant complications, CD grade III and above (10.0% vs 8.8%, *p* = 0.84), were equal between the two-stage and single-stage approach. The grades of the CD classification for both groups are summarised in Table 3.

**Table 3:** Postoperative complications categorised by Clavien-Dindo classification [25, 26] for the two-stage (intra-gastric balloon followed by laparoscopic Roux-en-Y gastric bypass) and single-stage (laparoscopic Roux-en-Y gastric bypass only) procedure

Clavien-Dindo (CD) classification, grade	Two-stage, *n* (%)	Single-stage, *n* (%)	*p* value
I	3 (10.0%)	0 (0.0%)	
II	1 (3.3%)	2 (1.6%)	
IIIa	0 (0.0%)	8 (6.4%)	
IIIb	2 (6.7%)	3 (2.4%)	
IVa	0 (0.0%)	0 (0.0%)	
IVb	0 (0.0%)	0 (0.0%)	
V	1 (3.3%)	0 (0.0%)	
Complications CD ≥ 3	3 (10.0%)	11 (8.8%)	0.84

## Discussion

LRYGB is a challenging operation in patients with BMI ≥ 50 kg/m^2^. This is the first study retrospectively treating these high risk patients using a multi-stage approach of IGB insertion followed by LRYGB surgery.

Preoperative co-morbidities were typical for individuals in this study and reflects on patient selection. Respiratory and cardiac co-morbidities, male sex were more frequently encountered in the two-stage group, whilst metabolic disorders such as diabetes were registered in a higher proportion of single-stage patients. High risk features were associated with higher BMI.

In terms of weight loss outcomes, there was improved weight loss with the single-stage LRYGB over the initial 6 months. However, there was no evident difference in weight loss between the two-stage and single-stage LRYGB approach at two years. Therefore, patients undergoing the two-stage approach may ultimately experience a delay in weight loss as the balloon remains in-situ for 6 months prior to the definitive procedure. This may in turn delay remission of diabetes mellitus and other metabolic co-morbidities.

In terms of safety and perioperative complications, high risk patients selected for two-stage approach had similar outcomes than lower risk patients undergoing the single-stage approach. There was no significant difference in length of stay following LRYGB between the two groups. Across all procedures, significant postoperative complications (higher than CD grade 3) were equal between high and moderate risk patients, suggesting that IGB prior to LRYGB may reduce subsequent perioperative morbidity. This may be due to a decrease in liver size and intra-abdominal adipose tissue following IGB insertion, which can reduce subsequent surgical complexity with less deviation from the planned approach [27, 28]. No major surgical complications, described in patients with obesity such as gastric perforation or necrosis [29], were encountered in this study.

There are non-invasive alternative methods to the IGB which must also be considered for patients which can achieve preoperative weight loss [29, 30]. Lifestyle modification and energy-restricted diet, or pharmacotherapy, can result in weight loss with a limited use of resources and fewer, if any, complications [31–33]. For example, glucagon-like peptide-1 (GLP-1) agonists, such as liraglutide and semaglutide, play an important role in central and peripheral modulation of appetite and body weight regulation. They have been shown to be an effective perioperative therapeutic option for weight loss, remission and improvement of obesity-related co-morbidities [34–36].

**Fig. 2:**
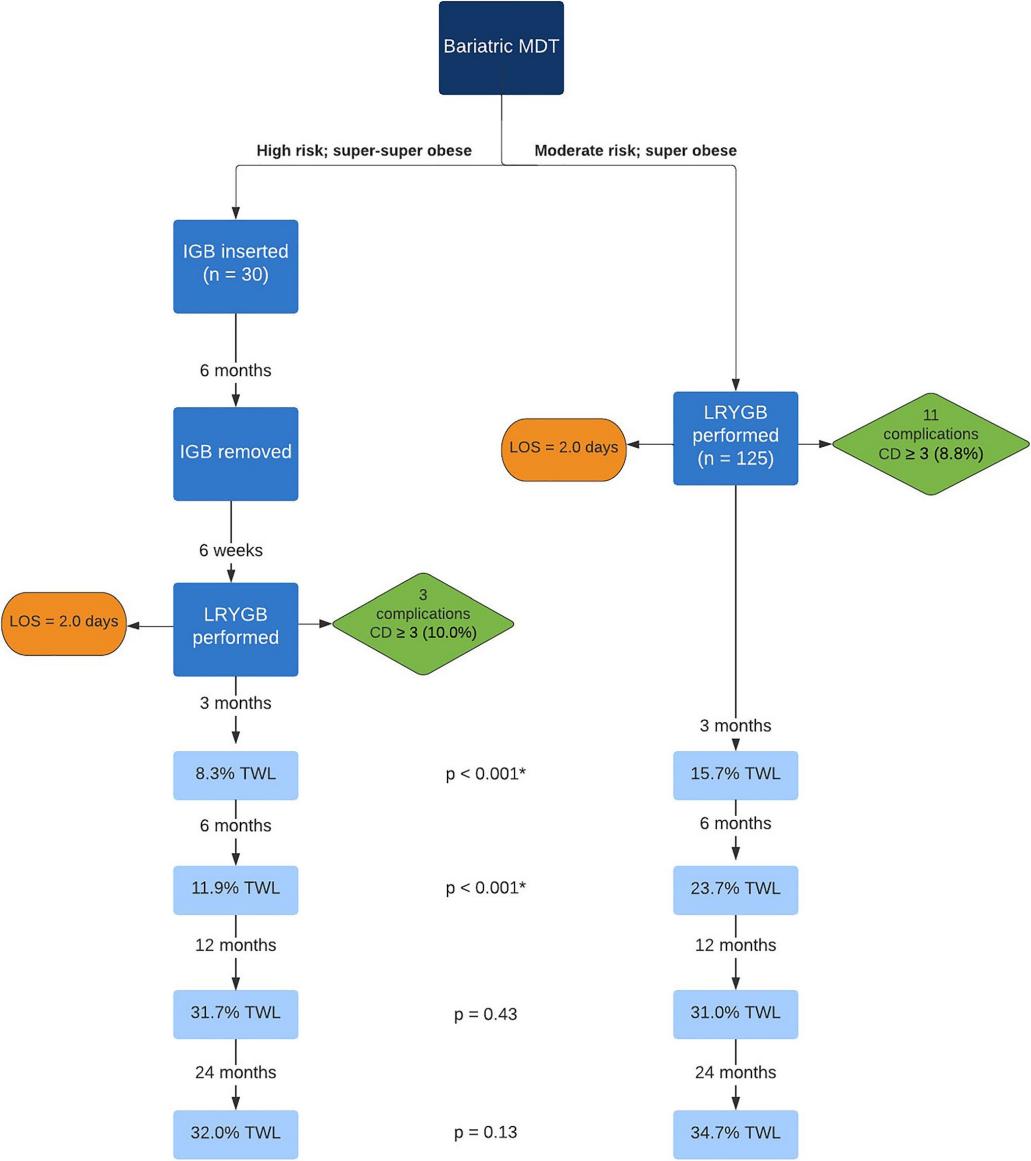
Flowchart of our retrospective study findings for the treatment of patients with obesity, BMI ≥ 50 kg/m^2^.* CD* Clavien-Dindo, *IGB* intra-gastric balloon, *LOS* length of stay, *LRYGB *laparoscopic Roux-en-Y gastric bypass, *MDT* multidisciplinary team, *TWL *total weight loss, * statistically significant

Our study demonstrated that IGB insertion leads to equal clinical outcomes in high risk patients as compared to moderate risk patients with obesity (BMI ≥ 50 kg/m^2^) following definitive LRYGB. The two-stage approach should be considered in these high risk patients. However, it is associated with a delay in weight loss; hence, moderate risk patients particularly with uncontrolled diabetes should proceed directly to LRYGB. A flowchart summarising the results of our retrospective study has been displayed in Fig. 2.

Early research suggested that initial IGB placement can act as a ‘kick starting’ procedure for short-term weight loss prior to definitive bariatric procedures such as LSG and laparoscopic gastric band insertion [21, 37]. Our findings suggest that two-year weight loss however cannot be enhanced with multi-stage procedure of IGB followed by LRYGB. Our data is consistent with the study presented by Banks et al. [38] for patients with BMI ≥ 50 kg/m^2^, where a small number of patients underwent a LSG rather than a LRYGB as a second procedure. They suggest that the use of IGB is not required as it is associated with delayed weight loss. In our study, which included a larger cohort of patients a delay in weight loss was also observed; however, besides equivalent weight loss we were able to demonstrate an improvement in clinical outcomes associated with the two-stage procedure.

It has been proposed that weight loss and metabolic enhancement may be greater in patients with type 1 diabetes mellitus who undergo definitive bariatric surgery; however, the mechanism beyond weight loss is yet to be fully understood. The current opinion is that immediate BRAVE (bile flow changes, restriction of stomach size, anatomic gastrointestinal rearrangement, vagal manipulation, enteric hormonal modulation) effects of surgery result in cascade effects on gut microbiome and local metabolism (intestinal gluconeogenesis and adipokine fluxes) [39].

Strengths of this study include a large cohort of patients with BMI ≥ 50 kg/m^2^ that underwent LRYGB with two-year follow-up, clear study design, rigorous statistical methods and robust data adjusted with propensity score weighting for baseline differences to reduce selection bias and to overcome confounding factors. In addition, all operations during the study period were performed by consultant surgeons with the same management protocols and standardised operative techniques.

However, we must accept that there are limitations associated with this study. The fact that the two cohorts had surgery at different time periods and lack of randomisation may have introduced some confounding factors. There were patients that were loss to follow-up or had incomplete data, and quality of life has not been assessed.

## Conclusions

The present study demonstrates equal weight loss outcomes after a single-stage approach for moderate risk and two-stage procedure for high risk patients with BMI ≥ 50 kg/m^2^ over a two-year period. The two-stage procedure of endoscopic IGB followed by LRYGB delays weight loss compared to the single-stage procedure. However, following the two-stage approach perioperative morbidity in high risk patients is equal to moderate risk patients undergoing definitive LRYGB which should be the primary treatment of choice for moderate risk patients. Randomised controlled trials comparing bariatric surgery with IGB insertion are required to further assess clinical outcomes in bariatric patients with a BMI ≥ 50 kg/m^2^.

## Supplementary Information

Below is the link to the electronic supplementary material.

Supplementary file1 (DOCX 111 KB)

## Data Availability

Registry used: clinicaltrials.gov. Unique Identifying number or registration ID: ClinicalTrials.gov Identifier: NCT05146011. Hyperlink to specific registration: https://clinicaltrials.gov/ct2/show/NCT05146011?cond=Clinical+Outcomes+of+Single+Stage+Versus+Two-stage+Laparoscopic+Roux-en-y+Gastric+Bypass+in+the+Management+of+Super+Obesity%3A+a+Propensity+Score+Weighted+Analysis&draw=2&rank=1

## Declarations

### Study design

Cohort study.
