# Supplementary material for: Clinical outcomes of single-stage versus two-stage laparoscopic Roux-en-y gastric bypass in the management of obesity (BMI ≥ 50 kg/m2): a retrospective cohort study
Source: Langenbecks Arch Surg. 2022 Sep 2;407(8):3349–56. doi: 10.1007/s00423-022-02664-9 (PMC9722810; doi:10.1007/s00423-022-02664-9)
Supplement: Supplementary file 1 — Supplementary file1 (DOCX 111 KB) [file 423_2022_2664_MOESM1_ESM.docx]

**Supplementary material**


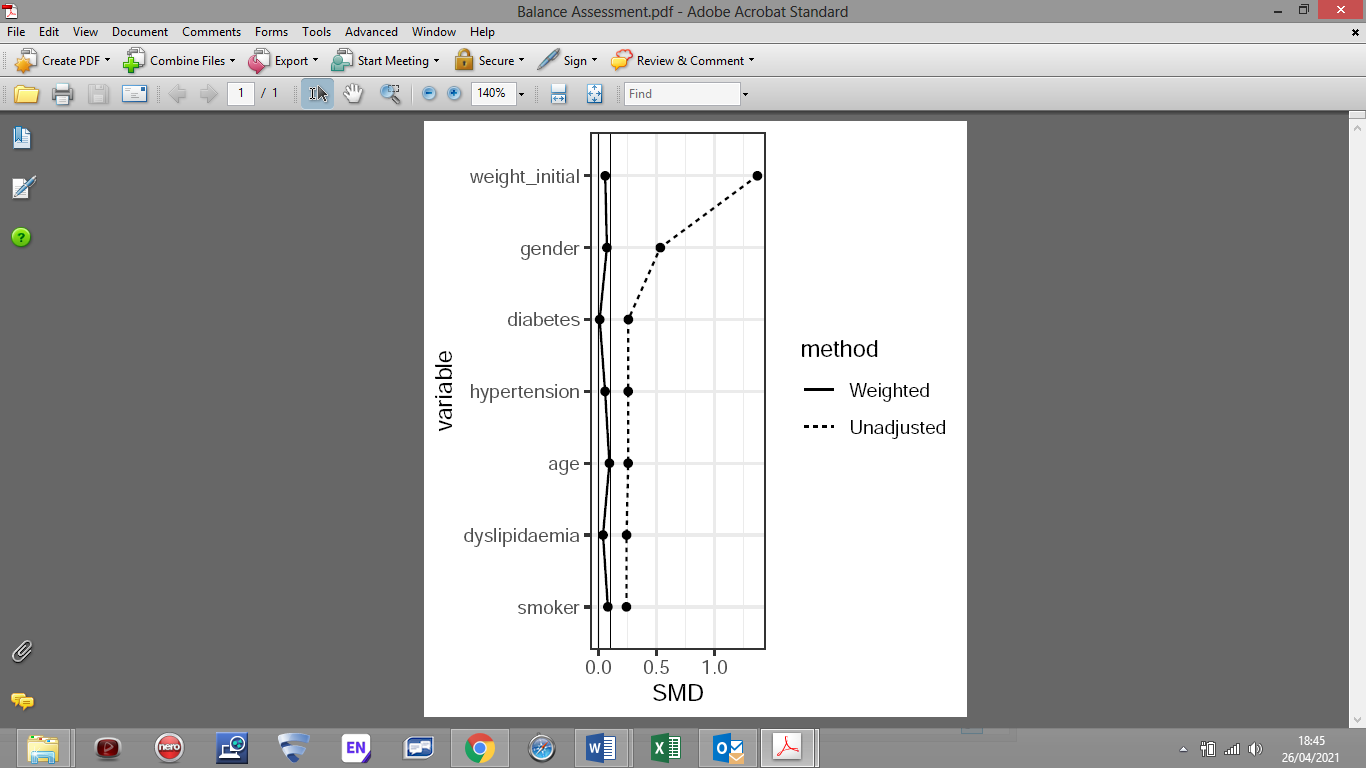


**Fig. S1.** Balance plot for weighted and unadjusted for all variables including initial weight, gender, dyslipidaemia, age, hypertension, diabetes and smoking. SMD = standardised mean difference.
